# Regulatory B cell-myeloma cell interaction confers immunosuppression and promotes their survival in the bone marrow milieu

**DOI:** 10.1038/bcj.2017.24

**Published:** 2017-03-24

**Authors:** L Zhang, Y-T Tai, M Ho, L Xing, D Chauhan, A Gang, L Qiu, K C Anderson

**Affiliations:** 1 LeBow Institute for Myeloma Therapeutics and Jerome Lipper Multiple Myeloma Center, Dana-Farber Cancer Institute, Harvard Medical School, Boston, MA, USA; 2 Department of Hematology, West China Hospital, Sichuan University, Chengdu, China; 3 UCD School of Medicine, College of Health and Agricultural Science and UCD Conway Institute of Biomolecular and Biomedical Research, University College Dublin, UCD, Dublin 4, Ireland; 4 State Key Laboratory of Experimental Hematology, Institute of Hematology and Blood Diseases Hospital, Chinese Academy of Medical Science and Peking Union Medical College, Tianjin, China

Multiple myeloma (MM), a terminally differentiated B-cell malignancy, is characterized by excess bone marrow (BM) plasma cells and immunosuppression. At present, the role of B-cell subsets in the MM-related immune suppressive BM microenvironment is not fully characterized. Regulatory B cells (Bregs), a small B-cell subset, can regulate immune responses via stimulation of anti-inflammatory cytokine interleukin 10 (IL10), and modulation of CD4^+^ T-cell activation and differentiation.^1^ In both animal models and man, Bregs in peripheral blood (PB) have been identified in the setting of autoimmune diseases, chronic inflammatory conditions and graft vs host disease post-allogeneic transplantation.^2, 3^ At present, the role of Bregs in cancers, including MM, has not been fully studied.^4^ Here we phenotypically define Bregs in MM and examine their role in mediating immunosuppression, which is a hallmark of this disease. The detailed methods are described in the Supplementary Material.

In autoimmune and other inflammatory conditions, human Bregs in PB are identified based on the CD19^+^CD24^high^CD38^high^ cell surface phenotype, whereas naive B cells are CD19^+^CD24^int^CD38^int^ and memory B cells are CD19^+^CD24^−^CD38^low/-^CD27^+^.^5, 6^ In this study, we characterized CD19^+^CD24^high^CD38^high^ Bregs in paired BM and PB samples from MM patients by flow cytometry. BM-, but not PB-, derived Bregs (CD19^+^CD24^high^CD38^high^) are a distinct subpopulation from the remaining mononuclear cells (Figures 1a and 2a; Supplementary Figure S2a). In contrast, the PB-derived Breg subset is not completely separated from the rest of PB mononuclear cells (PBMCs) as a detached population, consistent with previous reports where only blood samples from different autoimmune diseases were studied.^5, 6, 7^ Importantly, this is the first report that CD19^+^CD24^high^CD38^high^ Bregs are easily distinguished from other cells in the BM, but not PB, compartments from the same MM patient.

To determine the relationship between Bregs and disease status in MM, we next determined frequencies of Bregs within CD19^+^ B cells in MM at diagnosis, during maintenance treatment after MM response, and at time of relapse, in BM and PB samples. Frequencies of Bregs in CD19^+^ B cells in BM are significantly higher compared with PB from NDMM (14.04±1.77% vs 4.78±0.98% *P*<0.0005; Figure 1b). BM-derived Bregs within CD19^+^ B cells are significantly higher in patients with NDMM than in those on maintenance therapy after response (14.04±1.77% vs 1.48±0.83%, respectively, *P*<0.0001, *n*=10 for each group; Figure 1b). In a similar fashion, frequencies of PB-derived Bregs were significantly altered (5.04±1.97% vs 1.95±0.86%) in NDMM vs patients on maintenance therapy after response (*P*<0.02). At the time of MM relapse, CD19^+^ B cells, as well as Bregs in BM and PB, are too low to be detected. Finally, there are no significant differences in peripheral Bregs in NDMM vs normal donors (5.44±1.97% vs 4.56±0.86%). These results suggest that Bregs and patient cells may be dependent on each other in the BM microenvironment.

We next examined the function of BM-derived MM Bregs. Within NDMM BM, an increase in IL10 production is seen following stimulation of Bregs with lipopolysaccharides and phorbol 12-myristate 13-acetate (Figure 1a, right panel). The fraction of stimulated vs unstimulated BM-derived Bregs producing IL10 is 23.90±8.32 vs 1.5±0.07%, respectively. These results are in accord with previous studies,^1, 8^ and indicate that IL10-independent mechanisms are also operative mediating Breg function.

To further examine the novel function of Bregs, we asked whether Bregs modulate antibody-dependent cellular cytotoxicity (ADCC) against patient MM cells via natural killer (NK) cells. We purified NK effector cells from PBMCs and purified Bregs and naive B cells from NDMM BM following isolation of CD138 patient MM cells (*n*=3; Figures 1c and d; Supplementary Figure S1a–c). Cells were incubated for 4 h at effector (NK cells or PBMCs) to patient MM cell ratio of 4 to 1, in the presence or absence of Bregs or naive B cells, and with anti-SLAMF7/CS1 elotuzumab^9^ or isotype control IgG_1_ monoclonal antibodies. Lysis and killing of target MM cells were determined by staining with annexin V and zombie aqua, followed by flow cytometry analysis gated on carboxyfluorescein succinimidyl ester-labeled MM cells. Elotuzumab, but not isotype IgG_1_ control mAb (Figures 1c–4; Supplementary Figure S1c), significantly reduced viability of MM cells by approximately two-fold in the presence vs absence of NK cells, as shown in a representative sample (MM cell viability of 32.9 vs 67.8% in Figure 1c–1 with NK cells vs Figure 1c–3 without NK cells). Importantly, NK-mediated MM cell lysis triggered by elotuzumab is completely blocked by Bregs (Supplementary Figures 1c–2) and the viability of Bregs (~90%) remains unchanged in the end of ADCC assays (Supplementary Figure S1a). SLAMF7, the target antigen for elotuzumab, is undetectable on Bregs (*n*=6) (Supplementary Figure S1b), excluding potential NK killing of Bregs triggered by elotuzumab. The results from three samples shown in Figure 1d indicate that Bregs significantly reduce NK-mediated patient MM cell lysis induced by elotuzumab. PBMCs did not induce significant elotuzumab-mediated MM cell lysis at low E/T ratio, further confirming that NK cells are the key effector cells to lyse MM cells.^9^ Furthermore, Bregs, but not naive B subset, block NK cell-mediated ADCC against MM cells triggered by elotuzumab. The percentages of MM cell lysis by elotuzumab are significantly decreased (by 48±1.4 vs <1%) following addition of Bregs vs naive B subsets, respectively (Figure 1d). These results indicate that BM-derived Bregs, but not naive B subsets, are functionally effective in blocking ADCC induced by elotuzumab to lyse patient MM cells.

Having shown this inhibition of ADCC by MM BM-derived Bregs, we next examined the role of patient cells in modulating Bregs. Higher percentages of Bregs were found in the BM vs PB (10.3% vs 1.09%, respectively, Figure 2a upper flow panel). BM-derived Bregs were significantly (36%) decreased (from 12.7 to 8.12% within CD19^+^ lymphocytes) as early as 1 day following depletion of autologous CD138^+^ patient cells (CD138^−^ BM) in a representative NDMM patient. In contrast, both naive B and CD19^+^ B subsets remained unchanged after CD 138^+^ MM cell removal (Figure 2a lower flow panel, Figure 2b). As shown in a representative sample in the flow panel and table of Figure 2a, the percentages of naive B in CD19^+^ B cells are 3.59 and 3.49 in BM and CD138^−^ BM, respectively. In all seven MM patient samples studied, Bregs, but not naive B cells, are significantly reduced as early as 1 day after depletion of CD138^+^ patient cells (Figure 2b). Importantly, the percentage of Breg subsets within CD19^+^ B cells were also decreased in a time-dependent manner following the removal of autologous CD138^+^ myeloma cells (*n*=8, Figure 2c; Supplementary Figure S2a). Thus, the survival of Bregs, but not naive B cells, is dependent on MM cells in the BM.

To identify the mechanism whereby MM cells promote survival of Bregs, we next examined apoptosis of Bregs, either in the presence of CD138-depleted BM fraction alone or with add back of autologous CD138^+^ patient cells. Apoptotic Bregs within CD19^+^ B cells are significantly upregulated in the absence of autologous MM cells (CD138^−^ BM), whereas add back of CD138^+^ autologous MM cells reduces apoptosis and promotes survival of Bregs (Figures 2d, *n*=3; Supplementary Figure S2b).

In contrast to prior studies examining human Bregs circulating in PB and murine Bregs in spleens,^7^ the current report shows that immunosuppressive Bregs represent a distinct subset in MM BM. MM cells in the BM^10^ promote survival by inhibiting apoptosis of Bregs, which in turn mediates immunosuppression by production of IL10 and alternative mechanisms.^11^ Importantly, Bregs in the BM are upregulated at time of diagnosis and decreased at time of response and maintenance therapy. At time of relapse, CD19^+^ B cells, including Bregs, are too low to be detected.^12^ This is due in part to severe acquired immunodeficiency accompanying with a progressive depletion of lymphocytes, including CD19^+^ B cells, during relapse.

Finally, in the current era of immunotherapy for MM,^13, 14^ we here demonstrate that MM Bregs can abrogate NK cell-mediated ADCC against MM cells, further supporting Bregs as a novel cellular target of future therapeutics.^15^ Our current study also suggests that novel therapies targeting Bregs may enhance ADCC activity and MM cytotoxicity triggered by anti-SLAMF7, -CD38 and monoclonal antibodies against other MM target antigens.

In summary, our data show that MM BM Bregs confer an immunosuppressive BM microenvironment, which may in turn impact therapeutic response and disease outcome. Further understanding these unique B cell subsets will provide the rationale for targeting Bregs as a novel immuno-therapeutic strategy in MM.

## Figures and Tables

**Figure 1:**
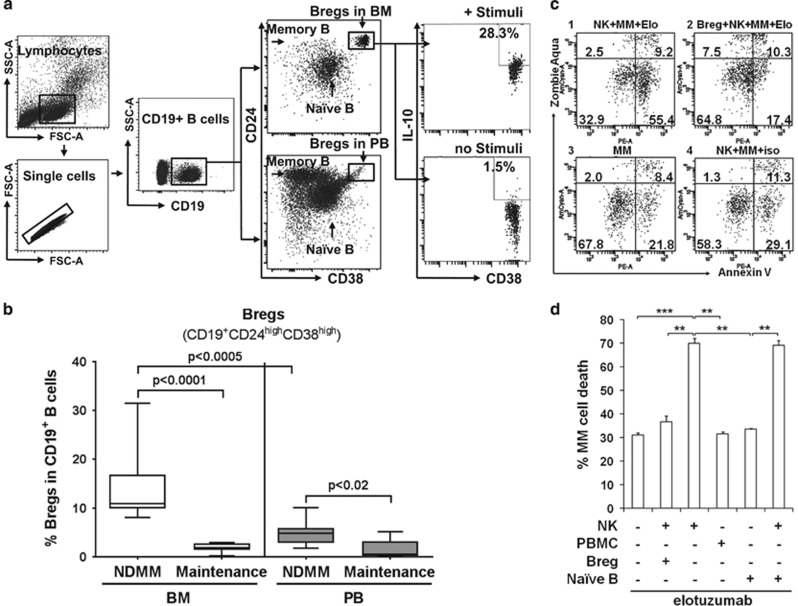
Regulatory CD19^+^CD24^high^CD38^high^ B cells with immunosuppressive properties are defined within BM more distinctly than PB in MM. (**a**) Bregs are phenotypically identified by flow cytometry as a distinct subset of CD19^+^CD24^high^CD38^high^ cells within BM, but not PB, from the same MM patient. Shown is a representative analysis of paired patient BM and PB with two separate B-cell populations: CD19^+^CD38^int^CD24^int^ B cells (primarily naive B cells) and CD19^+^ CD24^−^ CD38^low/−^ B cells (primarily memory B cells). BM-derived Bregs producing IL10 are significantly increased from 1.5 to 28.3% after stimulation with PMA and LPS (+ stimuli). (**b**) The percentages of BM-derived Bregs within CD19^+^ B cells are significantly higher in the NDMM group compared to the group who responded to treatment (maintenance) (*n*=10 for each group). (**c** and **d**) Bregs inhibit NK cell-mediated ADCC against MM target cells by elotuzumab. (**c**) Results of inhibition of BM-derived Bregs from a representative MM patient sample. 1, NK cells+MM cells+elotuzumab (elo); 2, BM-derived Bregs+NK cells+MM cells+elo; 3, MM cells alone; 4, NK cells+MM cells+isotype IgG_1_ control. (**d**) Shown are summary of % patient MM cell lysis in the presence or absence of BM-derived Bregs or naive B cells from NDMM (*n*=3), with or without effector cells. Data represent mean±s.d. for each group; **P<0.01, ***P<0.001, Student's *t*-test. FCM, flow cytometry; LPS, lipopolysaccharide; PMA, phorbol myristate acetate.

**Figure 2:**
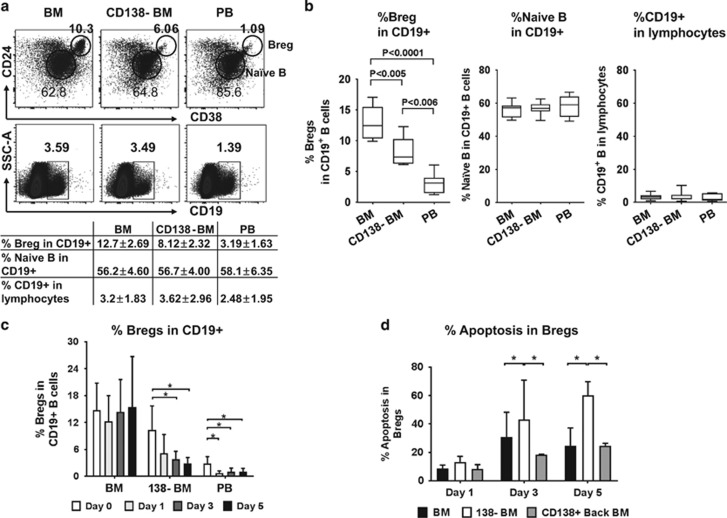
Myeloma cells promote survival and inhibit apoptosis of BM-derived Bregs from NDMM. (**a** and **b**) BM-derived Breg subset (CD19^+^CD24^high^CD38^high^) from NDMM patients (MM, *n*=7) are significantly decreased 1 day after depletion of CD138^+^ myeloma cells, whereas frequencies remain unchanged for naive B in CD19^+^ B cells and CD19^+^ B subset in lymphocytes. Shown are results from one representative NDMM (**a**) and summary of seven samples (**b**). CD138-BM, CD138-depleted bone marrow mononuclear cells (BMMCs). (**c**) The percentages of Bregs within CD19^+^ B cells (*n*=8) are determined 1, 3 and 5 days following depletion of CD138^+^ myeloma cells (CD138-BM). The percentages of Breg subset within CD19^+^ B cells significantly decreased in a time-dependent fashion. (**d**) The frequencies of apoptotic BM-derived Bregs in CD138-depleted BMMCs (CD138-BM) were significantly higher than in both freshly harvested BMMC (BM) and in CD138-depleted BMMCs with add back of CD138+ myeloma cells (CD138+ back BM) (*n*=3). Data represent mean±s.d.; *P<0.05, Student's *t*-test.

## References

[bib1] Rosser EC, Mauri C. Regulatory B cells: origin, phenotype, and function. Immunity 2015; 42: 607–612. 25902480 10.1016/j.immuni.2015.04.005

[bib2] Miyagaki T, Fujimoto M, Sato S. Regulatory B cells in human inflammatory and autoimmune diseases: from mouse models to clinical research. Int Immunol 2015; 27: 495–504. 25957264 10.1093/intimm/dxv026

[bib3] Chesneau M, Michel L, Degauque N, Brouard S. Regulatory B cells and tolerance in transplantation: from animal models to human. Front Immunol 2013; 4: 497. 24427159 10.3389/fimmu.2013.00497 PMC3876023

[bib4] He Y, Qian H, Liu Y, Duan L, Li Y, Shi G. The roles of regulatory B cells in cancer. J Immunol Res 2014; 2014: 215471. 24991577 10.1155/2014/215471 PMC4060293

[bib5] Blair PA, Norena LY, Flores-Borja F, Rawlings DJ, Isenberg DA, Ehrenstein MR et al. CD19(+)CD24(hi)CD38(hi) B cells exhibit regulatory capacity in healthy individuals but are functionally impaired in systemic Lupus Erythematosus patients. Immunity 2010; 32: 129–140. 20079667 10.1016/j.immuni.2009.11.009

[bib6] Czarnowicki T, Gonzalez J, Bonifacio KM, Shemer A, Xiangyu P, Kunjravia N et al. Diverse activation and differentiation of multiple B-cell subsets in patients with atopic dermatitis but not in patients with psoriasis. J Allergy Clin Immunol 2016; 137: 118–129, e115. 26441226 10.1016/j.jaci.2015.08.027

[bib7] Mauri C, Menon M. The expanding family of regulatory B cells. Int Immunol 2015; 27: 479–486. 26071023 10.1093/intimm/dxv038 PMC4587489

[bib8] Ray A, Wang L, Dittel BN. IL-10-independent regulatory B-cell subsets and mechanisms of action. Int Immunol 2015; 27: 531–536. 25999596 10.1093/intimm/dxv033 PMC11513724

[bib9] Tai YT, Dillon M, Song W, Leiba M, Li XF, Burger P et al. Anti-CS1 humanized monoclonal antibody HuLuc63 inhibits myeloma cell adhesion and induces antibody-dependent cellular cytotoxicity in the bone marrow milieu. Blood 2008; 112: 1329–1337. 17906076 10.1182/blood-2007-08-107292 PMC2515112

[bib10] An G, Acharya C, Feng X, Wen K, Zhong M, Zhang L et al. Osteoclasts promote immune suppressive microenvironment in multiple myeloma: therapeutic implication. Blood 2016; 128: 1590–1603. 27418644 10.1182/blood-2016-03-707547 PMC5034739

[bib11] Schwartz M, Zhang Y, Rosenblatt JD. B cell regulation of the anti-tumor response and role in carcinogenesis. J Immunother Cancer 2016; 4: 40. 27437104 10.1186/s40425-016-0145-x PMC4950763

[bib12] Pratt G, Goodyear O, Moss P. Immunodeficiency and immunotherapy in multiple myeloma. Br J Haematol 2007; 138: 563–579. 17686051 10.1111/j.1365-2141.2007.06705.x

[bib13] Tai YT, Anderson KC. A new era of immune therapy in multiple myeloma. Blood 2016; 128: 318–319. 27445408 10.1182/blood-2016-06-719856

[bib14] Hoyos V, Borrello I. The immunotherapy era of myeloma: monoclonal antibodies, vaccines, and adoptive T-cell therapies. Blood 2016; 128: 1679–1687. 27506540 10.1182/blood-2016-05-636357

[bib15] Nouel A, Simon Q, Jamin C, Pers JO, Hillion S. Regulatory B cells: an exciting target for future therapeutics in transplantation. Front Immunol 2014; 5: 11. 24478776 10.3389/fimmu.2014.00011 PMC3897876

