# Supplementary material for: Regulatory B cell-myeloma cell interaction confers immunosuppression and promotes their survival in the bone marrow milieu
Source: Blood Cancer J. 2017 Mar 24;7(3):e547–. doi: 10.1038/bcj.2017.24 (PMC5380908; doi:10.1038/bcj.2017.24)
Supplement: Supplementary Information [file bcj201724x1.docx]

**Supplementary materials and methods**

**Cell culture and Patient materials**

Peripheral blood and bone marrow samples from patients were collected in heparinized tubes at specified time points including at diagnosis and during treatment. Patients met the International Myeloma Working Group (IMWG) Criteria for the Diagnosis of MM*.* Heparinized blood was also obtained from healthy donors. Peripheral blood mononuclear cells (PBMCs) were isolated from whole blood, using a Ficoll-Paque density gradient centrifugation and plated in RPMI 1640 media supplemented with 20% fetal bovine serum (FBS, Gibco). BM samples were processed in a similar way. Cell numbers were quantified by hemocytometer. Written informed consent was obtained in all cases according to the Declaration of Helsinki.

**Flow cytometry analysis and cell sorting**

The majority of samples were evaluated using real-time multi-fluorochrome flow cytometry within 8 hours after arrived at our lab. Human Fc block reagent (BD Biosciences) was used to prevent nonspecific antibody binding. Breg were identified by staining with anti-CD19 PB (BioLegend), anti-CD24 APC (BioLegend), and anti-CD38 PE-Cy7 (BioLegend). The LIVE/DEAD Fixable Aqua Dead Cell Stain Kit (Invitrogen) was used to identify viable cells, in conjunction with PE-Annexin V (BioLegend) to identify apoptotic cells. Analysis was performed using BD FACSCanto™ II and BD LSRFortessa™ flow cytometer. Breg sorting was done using a BD FACS Aria II SORP Cell Sorter, and data were analyzed using Flowjo software.

In some experiments, CD138-coated beads (Miltenyi Biotec) were used to purify myeloma cells from BM samples by positive selection, following the manufacturer's instructions. NK cells were enriched by depletion of non NK cells using a cocktail of biotin-conjugated antibodies and anti-Biotin MicroBeads (Miltenyi Biotec).

**IL10 production by intracellular cytokine staining**

For intracellular staining of IL10 production, PBMCs and BMMCs from BM samples of NDMM were resuspended (1 × 10^6^ cells/mL) in RPMI 1640 media containing 10 μg/ml lipopolysaccharides (LPS, Escherichia coli serotype 0111: B4; Sigma-Aldrich) and 50 ng/ml phorbol 12-myristate 13-acetate (PMA, Sigma-Aldrich) and incubated in 5% CO_2_ at 37°C for 24 hours. The secretion inhibitors brefeldin A (BFA; BD) and monensin (BD) were added 4 hours before staining, according to manufacturer's directions. After the viability staining, cells were then surface stained for the Breg markers. Stained cells were fixed and permeabilized using a Cytofix/Cytoperm kit (BD Biosciences) and stained intracellularly with anti-IL10 PE (BioLegend).

**Antibody-dependent cell-mediated cytotoxicity assays**

In ADCC assays, NK or PBMC effector cells, in the presence or absence of BM-derived Bregs from MM patients were incubated with (CFSE) (Life Technologies)-labeled CD138-purified MM patient cells (20,000 target cells/well in a U bottom 96-well plate) at a ratio of 4:1 (E:T) and in the presence of 10 μg*/*ml Elotuzumab (elo) or isotype control human IgG_1_ for 4 h at 37°C in RPMI1640 media supplemented with 10% FBS. MM cells were pre-incubated with elotuzumab for 20 min, and then stained with the LIVE/DEAD Fixable Aqua Dead Cell Stain Kit (Invitrogen) and PE-Annexin V (BioLegend), according to manufacturer's directions. Viable cells were both Annexin V and Aqua negative (Annexin V-Aqua-), and the percentage of cytotoxicity was calculated as 100 × (total CFSE^+^ cells - viable cells)/ (total CFSE^+^ cells). To exclude the possibility of elo-induced ADCC against Bregs, CFSE-negative fractions (non-myeloma cells) were gated within CD19-positive fractions which distinguish Bregs from NK cells. Viable Bregs were determined by negativity for both annexin V and Aqua. SLAMF7 (CS1) is undetectable on Bregs (n=6) by FCS.

**Statistical analysis**

The results were expressed as the mean ± SD. Comparisons between 2 groups were performed with Student's *t*-test. Multiple groups (≥3) were analyzed by one-way ANOVA, and paired groups were analyzed by two-way ANOVA or Student t test. Data were graphed and analyzed using GraphPad Prism 6.0. P < 0.05 was considered statistically significant.

**
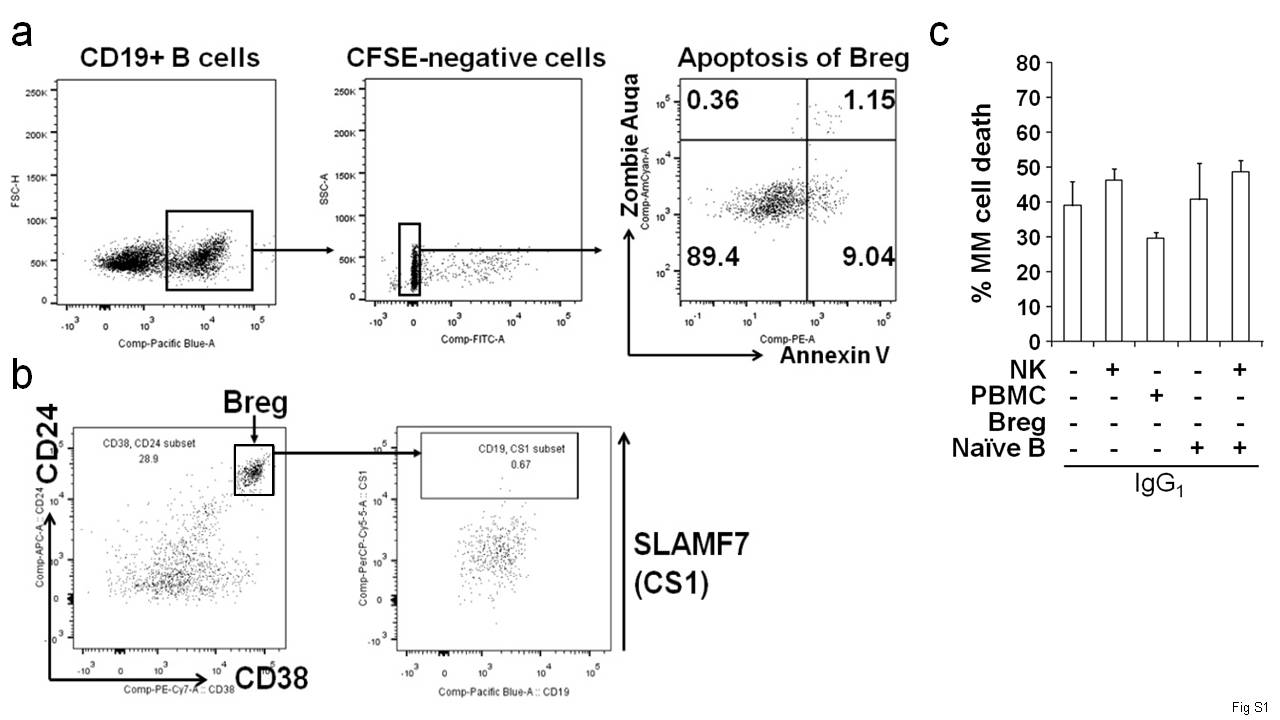
**

**Supplement Figure S1 BM-derived Bregs inhibit NK-mediated MM cell lysis induced by elotuzumab.** **a,** At the end of ADCC in Figure 1c, viability of Bregs were determined as percentages of negativity for both annexin V and zombie aqua after gating on CFSE^negative^ (non myeloma cells) and CD19+ cells in the mixture of Breg + NK +MM cells. Viability of Bregs is unaltered during 4h ADCC assays. **b,** SLAMF7/CS1 is undetectable on the cell membrane of Bregs from a representative sample. It is hardly detectable on Bregs derived from 6 patient samples tested. **c,** Control isotype IgG_1_ did not induce lysis of MM cells(n=3) induced by NK effector cells.

**
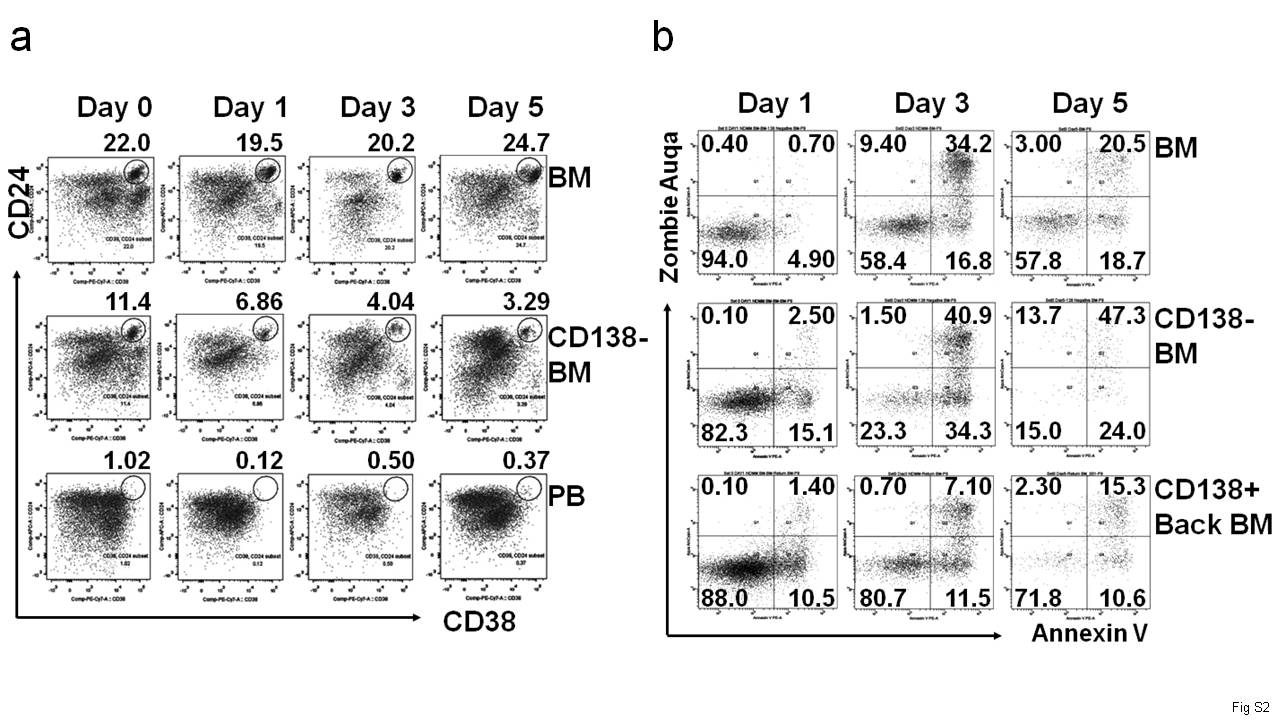
**

**Supplement Figure S2 The survival of Bregs, but not naïve B cells, is dependent on MM cells in the BM.**

**a**, The percentages of Bregs within CD19^+^ B cells are determined 1, 3, and 5 days following depletion of CD138^+^ myeloma cells (CD138- BM). The percentages of Breg subset within CD19^+^ B cells significantly decreased in a time-dependent fashion. Shown are data from a representative NDMM. **b**, The frequencies of apoptotic BM-derived Bregs in CD138-depleted BMMCs (CD138- BM) were significantly higher than in both freshly-harvested BMMC (BM) and in CD138-depleted BMMCs with add back of CD138+ myeloma cells (CD138+ back BM). Shown are results from 1 representative NDMM.
